# Why do zoos attract crows? A comparative study from Europe and Asia

**DOI:** 10.1002/ece3.5881

**Published:** 2019-11-26

**Authors:** László Kövér, Szabolcs Lengyel, Makiko Takenaka, Alice Kirchmeir, Florian Uhl, Rachael Miller, Christine Schwab

**Affiliations:** ^1^ Department of Nature Conservation Zoology and Game Management University of Debrecen Debrecen Hungary; ^2^ GINOP Sustainable Ecosystems Group Department of Tisza Research Danube Research Institute Centre for Ecological Research Hungarian Academy of Sciences Debrecen Hungary; ^3^ Department of Biology Tokai University Sapporo Campus Sapporo Japan; ^4^ Department of Cognitive Biology University of Vienna Vienna Austria; ^5^ Department of Psychology University of Cambridge Cambridge UK

**Keywords:** adaptation, artificial food, city planning, Corvidae, pest management, urban ecology

## Abstract

Crows have successfully colonized many cities, and urban zoos have been important in this process. To evaluate why zoos attract crows, we quantified crow numbers and behavior in three zoos in Europe (Debrecen, Edinburgh, Vienna) and one in Asia (Sapporo). Data were collected in 445 surveys over 297 days in summer 2014 and winter 2014–2015. We found that crow numbers were highest in Vienna, intermediate in Debrecen and Edinburgh and lowest in Sapporo, increased significantly from summer to winter (Debrecen, Edinburgh, Vienna), and from mornings to afternoons (Debrecen, Sapporo, Vienna), and were higher in sunny weather than in cloudy weather with precipitation and when visitor numbers were low (Debrecen, Vienna). The crows' use of natural food was highest in Vienna, intermediate in Edinburgh and Sapporo, and low in Debrecen. The use of anthropogenic food was high in Debrecen and Sapporo, where the availability of open grassy areas typically used by crows for natural foraging was low. In Sapporo, food availability was more limited than in other zoos, resulting in strong territoriality and few crows in summer, which decreased further in winter. Our study indicates that crows are primarily attracted to zoos by food availability and secondarily by breeding opportunities and that the relative importance of natural versus anthropogenic food sources may vary with zoo habitat structure. Our study draws attention to a previously overlooked role of zoos in urban biodiversity conservation. It may also provide useful information for the management of crow populations, if necessary, and for the planning of urban areas.

## Graphical abstract

Crows are primary attracted to zoos by food availability and secondly by breeding opportunities. Crows' number increased significantly from summer to winter and from mornings to afternoons. The number of visitors influenced crow numbers as well as the availability of food. The structure of zoos and food management affected the appearance of crows. Zoos could play important role regarding to crow control.

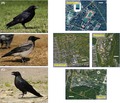

## 1 INTRODUCTION

Many species have adapted to human‐transformed landscapes and some have even become dependent on urban resources (Kark, Iwaniuk, Schalimtzek, & Banker, 2007; Marzluff et al., 2008; Preininger, Schoas, Kramer, & Boeckle, 2019; Vuorisalo, Talvitie, Kauhala, Bläuer, & Lahtinen, 2014). Cities are a special ecosystem for their abiotic and biotic characteristics and their unique species composition (Bezzel, 1985; Fey, Vuorisalo, Lehikoinen, & Selonen, 2015; Parlange, 1998). Urbanization usually results in decreased diversity and more homogeneous composition of bird species (Crooks, Suarez, & Bolger, 2003; Jokimäki & Suhonen, 1993), leading to both biotic (Devictor et al., 2008) and phylogenetic homogenization (Morelli et al., 2016). Urban environments, however, also provide benefits to certain species, for example, several species in the bird family Corvidae successfully colonized urban landscapes across the world (Cramp & Perrins, 1994). Particularly, successful colonizing species include the Hooded Crow (*Corvus cornix*) (Kövér et al., 2015; Vuorisalo et al., 2003) and the Eurasian Magpie (*Pica pica*) (Jerzak, 2001; Jokimäki, Suhonen, Vuorisalo, Kövér, & Kaisanlahti‐Jokimäki, 2017; Vuorisalo, Hugg, Kaitaniemi, Lappalainen, & Vesanto, 1992) in Europe, the American Crow (*C. brachyrhynchos*) (Marzluff, McGowan, Donnelly, & Knight, 2001) in North America, and the Large‐billed Crow (*C. macrorhynchos*) (Takenaka, 2003; Ueta, Kurosawa, Hamao, Kawachi, & Higuchi, 2003) in eastern Asia. Corvids are known for their behavioral plasticity and intelligence, which enable them to readily adapt to new environments, including artificial environments constructed by humans (Bird & Emery, 2009; Emery & Clayton, 2004; Hunt, 2014; Marzluff et al., 2008). Some crows are thus considered as “urban exploiters” (Japanese Ministry of Environment, 2001; Kark et al., 2007; Nishikawa, 2004) or even as nuisance or pest animals that feed on trash and spread diseases (Preininger et al., 2019).

The benefits that urban environments provide to bird species include the year‐round availability of food, milder local climate, decreased or no predator pressure, and diverse nesting opportunities (Eötvös, Magura, & Lövei, 2018; Marzluff et al., 2001; Vuorisalo et al., 2003). An understanding of the relative importance of these factors, however, is difficult due to the variation in urban environments in terms of human population size, the built‐in and natural components of urban landscapes, and type and availability of food sources. A comparison of corvid use of urban landscapes that are similar in human population size, urban landscape composition, and food availability can thus provide insights into the relative importance of the factors suspected to explain corvid colonization of urban environments (Preininger et al., 2019).

Zoos offer a great possibility for such comparisons. Zoos are found in many cities of the world and are typically viewed as cultural landscapes (Axelsson & May, 2008; Hallman & Benbow, 2006) that often show similarities in area (up to few tens of hectares), landscape composition (parks with trees and open spaces), and food availability to free‐living birds (food given to zoo animals, leftover/garbage from humans). Zoos that allow access to free‐living birds and that provide permanent sources of food may be the first to be colonized by corvids in a city and are usually characterized by high nesting density (Kövér et al., 2015). Zoos thus provide an excellent opportunity to study the factors that influence the establishment and colonization of corvids in urban environments (Uhl et al., 2018). However, to our knowledge, there is no study specifically investigating the role that zoos play in the colonization of cities by free‐living birds. We thus know little on why zoos attract free‐living birds such as crows, that is, how crows use the zoos and what are the factors that may make zoos attractive to crows.

The aim of this study was to evaluate why crows are attracted to zoos, that is, the role that zoos play in the establishment and colonization of free‐living corvids in urban environments. We focused on food availability and breeding opportunities as factors that may explain the attractiveness of zoos to crows. We hypothesized that (a) crow abundance may vary between zoos with different geographic settings, habitat structures, and/or food management practices, (b) crow abundance may change between seasons, as we expected more crows in the winter (when food is generally limited and crows are gregarious) than in the summer (when food is usually not limited and crows are territorial), and (c) crow abundance may be higher in the afternoons, as zoo animals are typically fed during the day, for example, at scheduled feeding times for visitors, and by the afternoon, the leftovers will become available to crows. To study these factors, we quantified the number of corvids, including, as far as possible breeding (i.e., resident) and nonbreeding corvids (i.e., nonresident), and recorded their behavior over one summer and one winter in four urban zoos that differed in geographic settings, habitat structures, and/or food management practices. We studied the effects of food availability, season, time of day, weather, temperature, and snow cover on corvid numbers. We also quantified crow behavior to evaluate the importance of natural versus anthropogenic food sources and breeding opportunities in order to detect differences between zoos that can be used to infer the importance of the above factors in corvid establishment and colonization of urban areas.

## 2 MATERIALS AND METHODS

### 2.1 Study locations and species

**Figure 1:**
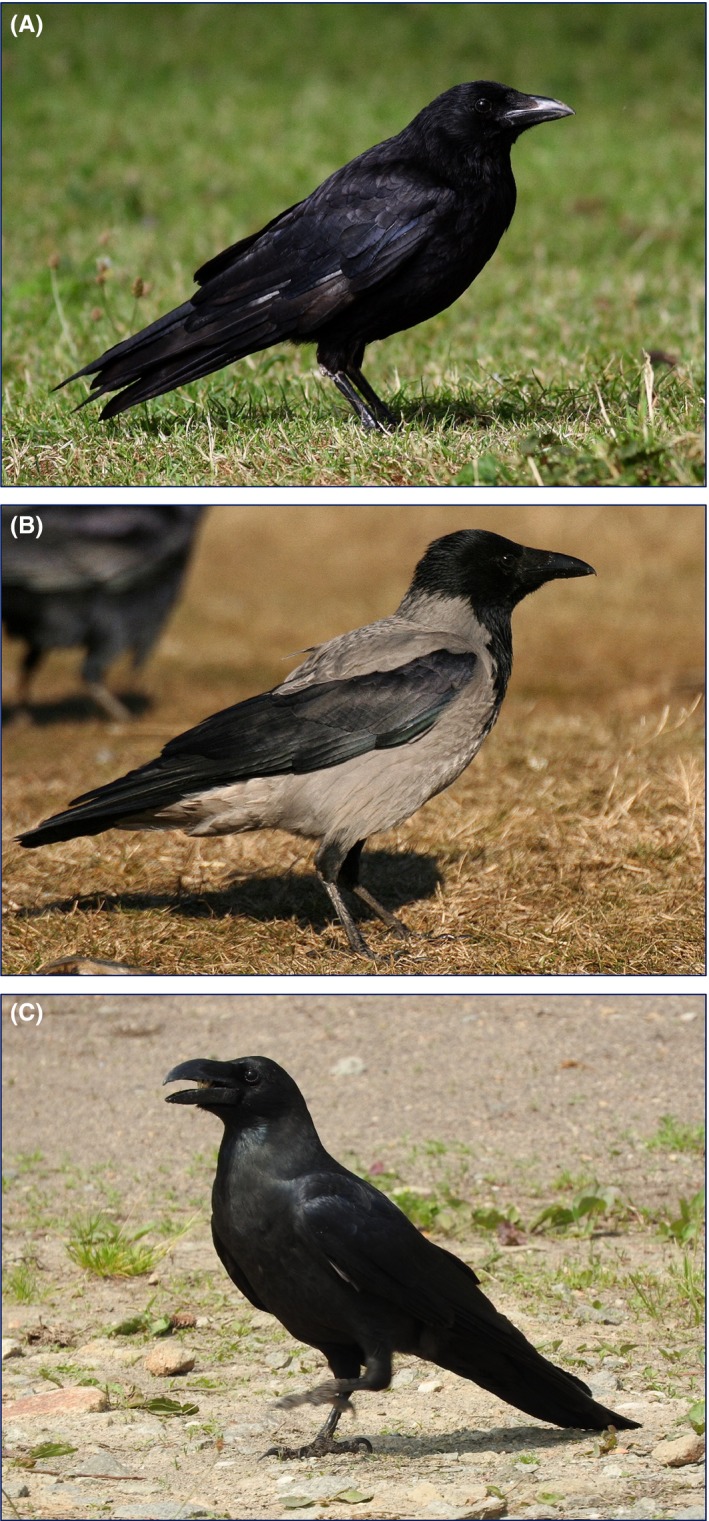
Photographs of crow species studied: Carrion Crow (*Corvus corone*) (A), Hooded Crow (*C. cornix*) (B), and Large‐billed Crow (*C. macrorhynchos*) (C). Photos by Péter Gyüre (A, B) and Makiko Takenaka (C)

**Figure 2:**
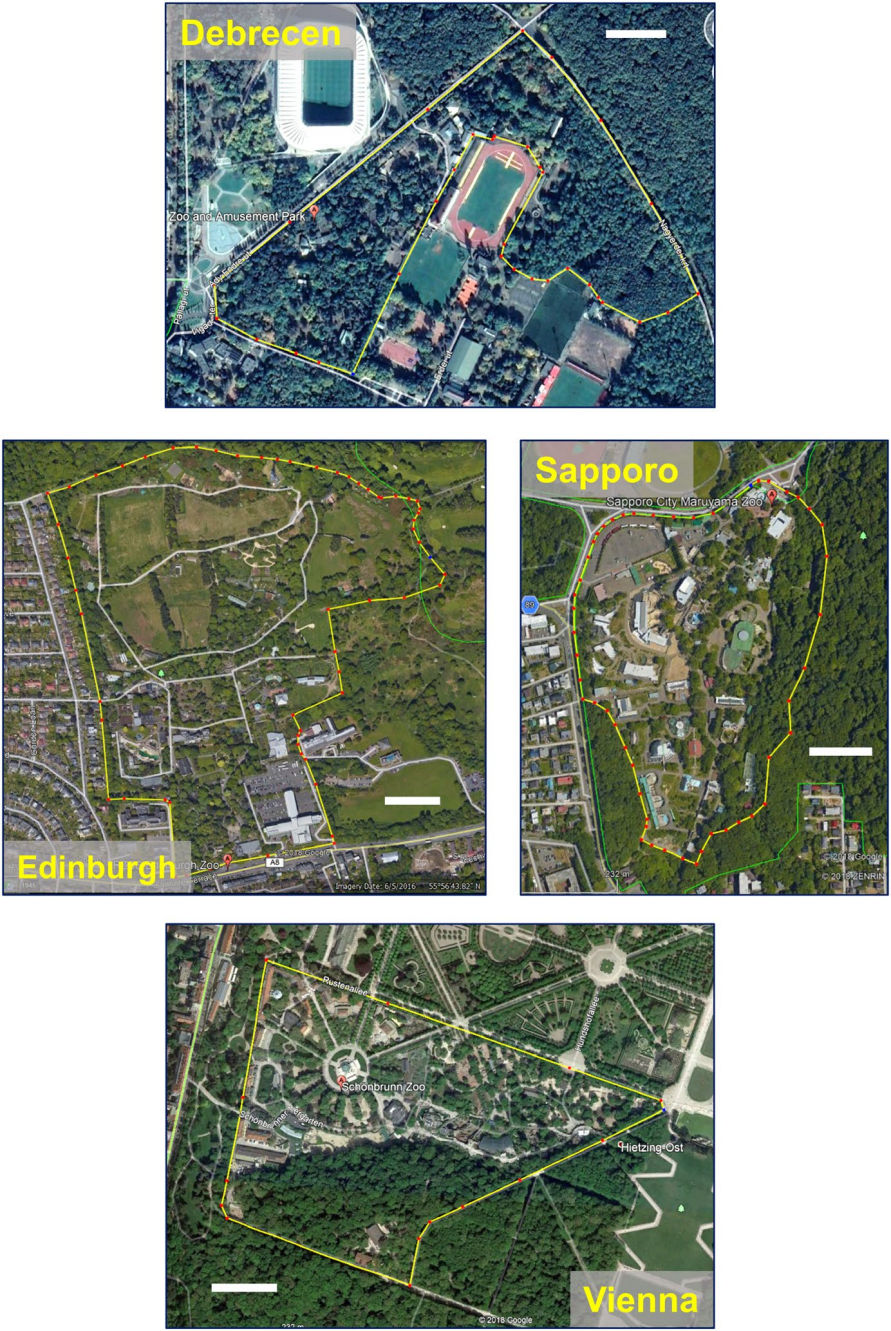
Aerial view of the four zoos (borders in yellow, white horizontal bars: 100 m). Source: Google Earth

We studied three crow species (Figure 1) in four zoos in Europe and Asia: Debrecen Zoo in Debrecen, Hungary, Edinburgh Zoo in Edinburgh, United Kingdom, Sapporo Maruyama Zoo in Sapporo, Japan, and Tiergarten Schönbrunn in Vienna, Austria (Table 1, Figure 2). Each zoo is within the respective city limits, displays several hundreds of animals in indoor and outdoor enclosures, and has both open habitats (e.g., grasslands, lawns) and woody vegetation, walkways, and facilities for visitors such as restaurants, kiosks, and outdoor eateries (Table 1, Figure 2). The zoos are inhabited and utilized by *Corvus* crow species: two species of crows (carrion crows *C. corone* and hooded crows *C. cornix*) in the three zoos in Europe and large‐billed crows in Sapporo (Table 1). The two European species, *C. corone* and *C. cornix*, co‐occur and often form mixed breeding pairs in Vienna. Although other corvid species were also found occasionally in the zoos (European jackdaw *C. monedula* and rook *C. frugilegus* mainly in the winter in Debrecen and Vienna, European jackdaw and Eurasian magpie *Pica pica* in Edinburgh, carrion crow and Eurasian jay *Garrulus glandarius* in Sapporo), we focused our study on the most common crow species in each zoo (Table 1). The four zoos differed in geographic setting (one in northwest Europe, two in Central Europe, and one in Asia), food availability (Sapporo with fewer open‐top enclosures and strict food management practices, three others with more open‐top enclosures and less strict practices), number of visitors (Debrecen < Edinburgh = Sapporo < Vienna), and habitat structure (with fewer trees relative to area and more open habitat in Edinburgh and Vienna and relatively more trees and less open habitat in Debrecen and Sapporo) (Table 1).

**Table 1:** Characteristics of the studied zoos

City	Coordinates	Common *Corvus* species	Zoo area (ha)	% open‐top enclosures	*N* visitors (2013)	*N* potential nest trees	*N* breeding pairs (2014)
Debrecen	47°31′48″N, 21°38′21″E	Hooded Crow, *C. cornix*	17	67	172,500	c. 2,000	8
Edinburgh	55°56′35″N, 3°16′05″W	Carrion Crow, *C. corone*	33	69	760,897	c. 1,200	10
Sapporo	43°03′48″N, 141°20′55″E	Large‐billed Crow, *C. macrorhynchos*	22.5	38	748,819	c. 2,900	8
Vienna	48°10′56″N, 16°18′09″E	Carrion Crow, Hooded Crow	17	59	2,226,404	c. 1,700	21

### 2.2 Data collection

We collected data by recording the number and behavior of crows in the zoos in the summer of 2014 and winter of 2014/2015 (Table 2). We surveyed crows while walking along a predefined route that covered the entire zoo area. One or more surveys were conducted on every study day (mean 1.6 ± S.D. 0.37, range 1.2–2.7 surveys per day in the summer and 1.5 ± 0.16, range 1.2–1.9 surveys per day in the winter). The total number of study days was 85, 72, 82,and 58 in Debrecen, Edinburgh, Sapporo, and Vienna, respectively, and the number of surveys was 100, 88, 114, and 143, respectively (Table 2). On average, surveys were conducted once every 2.4 ± 0.17 days (range 2.1–2.9) in the summer and once every 2.5 ± 0.33 days (range 1.9–3.3) in the winter (all zoos combined).

**Table 2:** Characteristics of sampling (number of study days and surveys) in the summer and the winter periods in the four zoos

City	Summer (2014)	*N* days	*N* surveys	Winter (2014/15)	*N* days	*N* surveys
Debrecen	22 April to 17 August	55	63	5 December to 6 February	30	37
Edinburgh	21 May to 12 August	40	47	15 December to 13 February	32	41
Sapporo	16 April to 17 August	52	69	3 December to 28 February	30	45
Vienna	1 May to 22 August	40	108	2 December to 30 January	18	35

In each survey, we recorded the location of sighting for each crow and the behavior of the bird. We defined three categories of crow behavior: (a) “foraging” was searching for or consuming naturally occurring food on the ground, in the grass, or in the trees, (b) “feeding” was searching for or consuming food from anthropogenic sources either as food provided to the zoo animals, given directly to crows by humans or indirectly as leftover near restaurants, kiosks, or trash bins, and (c) “breeding” included all behaviors suggesting that an animal was breeding (collecting nest material, incubating eggs, guarding a nest, guarding or feeding young etc., only in summer). Surveys were conducted only during the time when zoos were open to the public. We noted the number of human visitors in the vicinity (<50 m) of each crow sighting and calculated the average number of visitors for each survey. The number of visitors was then classified into three categories as few (between 1 and 5 visitors), intermediate (between 6 and 10 visitors), and many (>10 visitors). Finally, we recorded season (summer, winter), time of day (morning, afternoon, with threshold of 13:00 in summer and 12:00 in winter), weather conditions (sunny, cloudy, cloudy with precipitation such as rain or snow), air temperature (measured by handheld thermometers), and presence of snow cover (only in winter). Air temperature measurements were classified into three categories (cold: <10°C, moderate: between 10°C and 20°C, warm: >20°C). No surveys were conducted in heavy rain or snow or when air temperature was below −10°C or above 30°C.

### 2.3 Data analyses

Our first response variable was the number of observed crows per survey. To correct for the different area of zoos, we calculated this as the number of crows per 10 ha. We evaluated the effect of predictor variables on the response variable by constructing a generalized linear mixed model (GLMM) with a log‐linear link function and a Poisson error distribution. For the GLMM, we pooled summer and winter data, and “day” was entered into the model as random factor to account for repeated measures, that is, more than one survey on the same day and to minimize the effect of temporal autocorrelation in the observations. Fixed main effects included zoo (Debrecen, Edinburgh, Sapporo, Vienna), season, time of day, weather, temperature, snow cover, and number of visitors. We did not differentiate between observations on weekdays and weekends/holidays because the number of visitors was considered a more direct measure of human presence in the zoos.

Because our primary interest was to find between‐zoo differences (i.e., interactions) and similarities (i.e., lack of interactions) in the effect of predictor variables, we entered all first‐order interactions with “zoo” to allow for different relationships with fixed effects in the four zoos (full model). Nonsignificant (*p* < .05) interactions and main effects were removed sequentially to obtain a final reduced model. When any interaction with “zoo” was significant, we applied pairwise comparisons of zoos using *t* tests, with a significance level corrected for multiple comparisons (Bonferroni *α* = .005). To confirm our final model, we also carried out model selection in an information theory‐based approach based on AICc using the “dredge” function of the R package “MuMin.” This analysis resulted in one best model (for the second best model, ΔAICc = 2.218 or >2), which was identical to our final model; thus, we concluded that an alternative approach would lead to qualitatively identical results.

Our second response variable was crow behavior. To compare crow behavior within and between zoos, we calculated percentages of the three behavioral variables separately for each zoo as the percentage of occasions when birds were observed performing any of the three behaviors in each survey. As we only recorded foraging, feeding, and breeding and crows could engage in other behaviors, these percentages did not necessarily add up to 100% in each survey. We compared behavioral data (percentages) between and within zoos separately for seasons. As the behavioral data were not normally distributed, we used Kruskal–Wallis and Mann–Whitney *U* tests for between‐zoo comparisons. In pairwise Mann–Whitney *U* tests, we applied Bonferroni adjustment for multiple comparisons by setting *α* to .008 (between‐zoo comparisons) or to .016 (within‐zoo comparisons). Significance values given are two‐tailed, and all analyses were performed with IBM SPSS Statistics 20.

## 3 RESULTS

### 3.1 Factors influencing the number of crows

**Figure 3:**
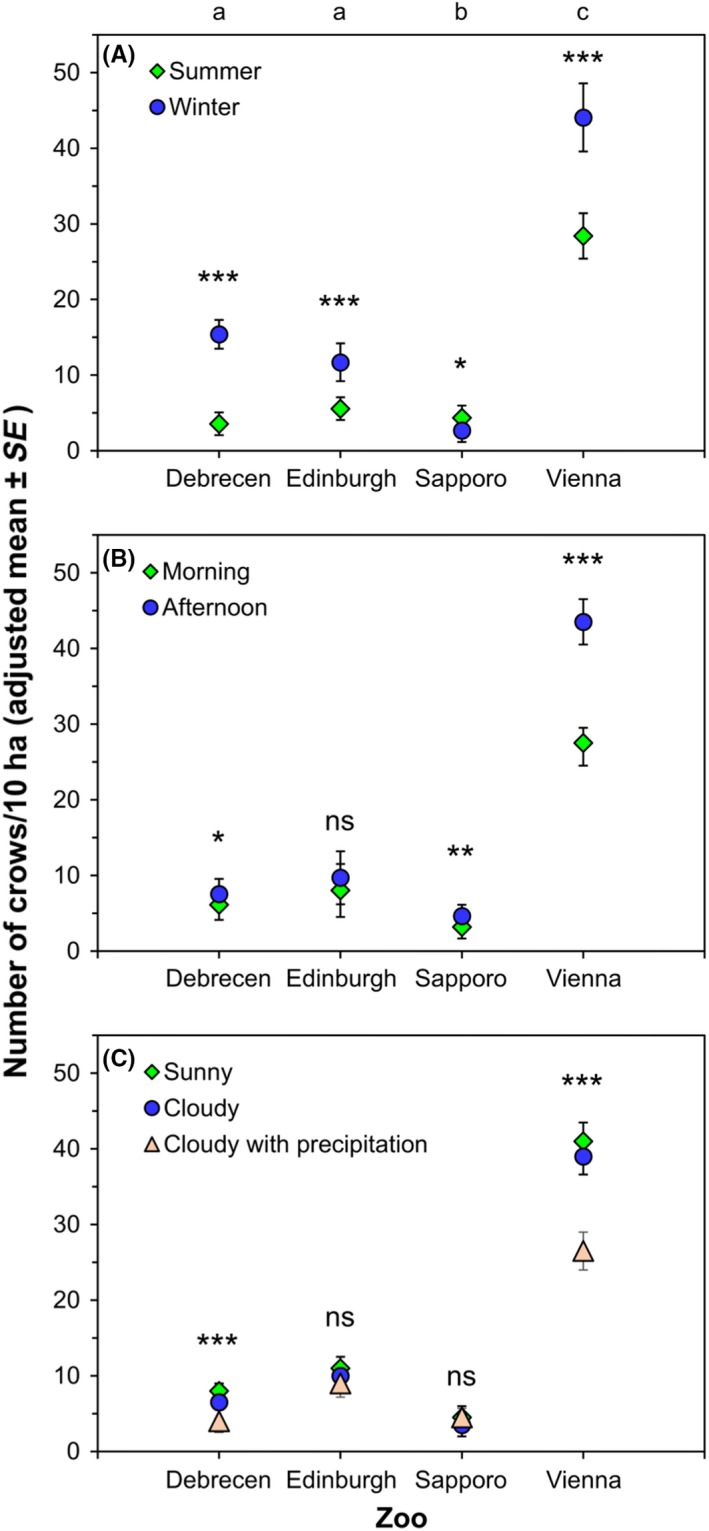
Mean ± *SE* number of crows observed in the four zoos in summer and winter surveys (A), in morning and afternoon surveys (B), and in surveys in sunny, cloudy weather, and cloudy weather with precipitation (C). Values shown are means adjusted for fixed and random effects by the final GLMM. Lowercase letters above (A) indicate significant differences (*p* < .01) in the number of crows in the between‐zoo comparison, with data pooled across season, time of day and weather. Asterisks indicate results of within‐zoo comparisons, *p < .05, **p < .01, ***p < .001, ns: not significant

We observed significantly more crows in Vienna than in the other three zoos (Figure 3; Vienna–Edinburgh: *t* = 10.145, *df* = 415, *p* < .001; Vienna–Debrecen: *t* = 13.526, *df* = 415, *p* < .001; Vienna–Sapporo: *t* = 15.103, *df* = 415, *p* < .001), while the number of crows was intermediate and similar in Debrecen and Edinburgh and was significantly lower in Sapporo than in Debrecen or Edinburgh (Debrecen‐Sapporo: *t* = 3.801, *df* = 415, *p* < .001; Edinburgh‐Sapporo: *t* = 3.101, *df* = 415, *p* = .002) (Figure 3, Table 3).

**Table 3:** Results of generalized linear mixed models testing the effects of fixed effects on the number of crows observed per survey with day of observation as random factor

Fixed factor	Full model			Final model		
	*F*	*df*1, *df*2	*p*	*F*	*df*1, *df*2	*p*
Zoo identity	55.238	3, 405	<.001	**164.717**	**3, 415**	**<.001**
Season	3.076	1, 405	.08	**76.164**	**1, 415**	**<.001**
Time of day	36.769	1, 405	<.001	**39.228**	**1, 415**	**<.001**
Weather	4.583	3, 405	.004	**5.273**	**3, 415**	**.001**
Temperature	0.088	2, 405	.916			
Snow cover	0.638	1, 405	.425			
Number of visitors	4.843	2, 405	.008	**4.586**	**2, 415**	**.011**
Zoo*Season	6.094	2, 405	.002	**28.208**	**3, 415**	**<.001**
Zoo*Time of day	4.387	3, 405	.005	**4.537**	**3, 415**	**.004**
Zoo*Weather	3.698	7, 405	.001	**3.483**	**7, 415**	**.001**
Zoo*Temperature	0.662	5, 405	.653			
Zoo*Snow cover	1.158	2, 405	.315			
Zoo*Number of visitors	2.566	6, 405	.019	**3.006**	**6, 415**	**.007**

Note

The full model contained zoo identity, six fixed effects, and their six first‐order interactions with zoo identity, whereas the final model contained only significant (*p* < .05) factors and interactions (indicated in bold).

Significant interactions between zoos and four factors showed that the effects of season, time of day, weather, and number of visitors on the number of crows differed between the zoos (Table 3). The interaction between zoo and season was because there were more crows in the winter than in the summer in Debrecen (*F*_1, 415_ = 68.181, *p* < .001), Edinburgh (*F*_1, 415_ = 17.068, *p* < .001), and Vienna (*F*_1, 415_ = 17.47, *p* < .001), whereas we found the opposite in Sapporo (*F*_1, 415_ = 6.589, *p* = .011) (Figure 3A, Table 3). The interaction between zoo and time of day indicated that there were more crows in the afternoon than in the morning in Debrecen (*F*_1, 415_ = 5.439, *p* = .02), Sapporo (*F*_1, 415_ = 9.743, *p* = .002), and Vienna (*F*_1, 415_ = 128.526, *p* < .001), whereas a similar difference in Edinburgh was not significant (*F*_1, 415_ = 0.938, *p* = .333) (Figure 3B, Table 3). The interaction between zoo and weather indicated that there were more crows in sunny weather than in cloudy weather with precipitation in Debrecen (*F*_2, 415_ = 9.531, *p* < .001) and Vienna (*F*_2, 415_ = 18.829, *p* < .001), whereas there were no such differences in Edinburgh (*F*_2, 415_ = 1.008, *p* = .389) and Sapporo (*F*_2, 415_ = 2.49, *p* = .084) (Figure 3C, Table 3). Finally, the interaction between zoo and the number of visitors was because there were more crows when there were a few or some visitors than when there were many visitors in Debrecen (few vs. many: *t* = 3.881, *df* = 415, *p* < .001; some vs. many: *t* = 3.995, *df* = 415, *p* < .001), whereas there were more crows when there were some visitors compared to few or many visitors in Vienna (few vs. some: *t* = 4.709, *df* = 415, *p* < .001; some vs. many: *t* = 2.657, *df* = 415, *p* = .008). The number of crows observed was not related to the number of visitors in Edinburgh (*F*_2, 415_ = 0.1, *p* = .905) and Sapporo (*F*_2, 415_ = 2.722, *p* = .067).

### 3.2 Crow behavior differences between and within zoos

**Figure 4:**
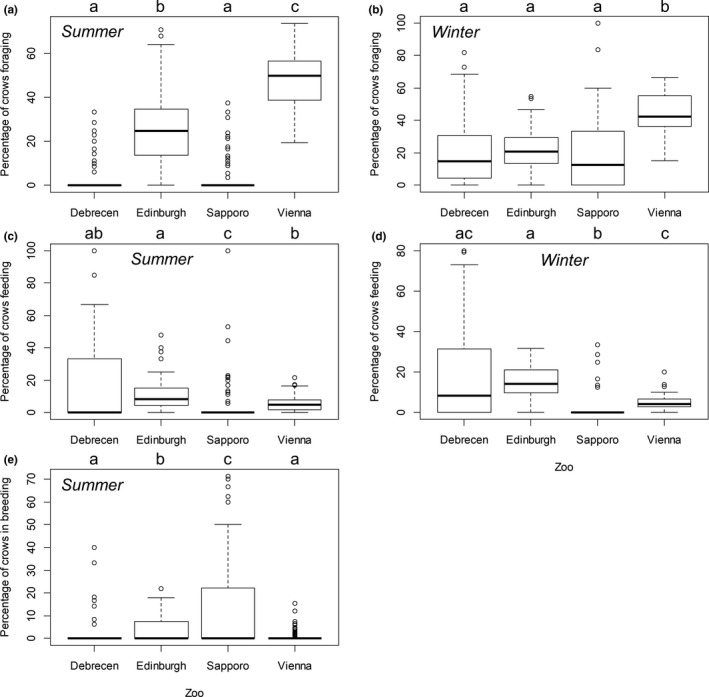
Percentage of crows observed engaged in foraging (natural food, A, B), feeding (anthropogenic food, C, D), and breeding (E) behavior in the summer (left‐hand column) and winter (right‐hand column) in the four zoos. Lowercase letters above the graphs indicate significant differences between zoos (pairwise Mann–Whitney tests, Bonferroni‐adjusted two‐tailed probabilities, *p* < .008)

Between‐zoo comparisons revealed significant variation in each of the three crow behaviors recorded between zoos (Figure 4). In summer, the frequency of foraging (use of natural food sources) was highest in Vienna, followed by Edinburgh, and was low in Debrecen and Sapporo (Figure 4A, Table 4). The frequency of feeding (use of anthropogenic food sources) was high (but also highly variable) in Debrecen, relatively high in Edinburgh, followed by Vienna, and was lowest in Sapporo (Figure 4C, Table 4). The frequency of breeding behaviors was highest in Sapporo, followed by Edinburgh, then by Vienna and Debrecen, with no significant difference between the latter two (Figure 4E, Table 4). In the winter, the frequency of foraging behavior was highest in Vienna and significantly lower (15%–20%) in the other three zoos, indicating a decrease from summer levels in Edinburgh and increases in Debrecen and Sapporo (Figure 4B, Table 4). The frequency of feeding behavior increased from summer to winter in Debrecen and Edinburgh and was significantly higher in the winter than in Sapporo and Vienna (Figure 4D, Table 4).

**Table 4:** Between‐zoo pairwise comparisons of foraging (use of natural food sources), feeding (use of anthropogenic food sources), and breeding behaviors in summer (data in Figure 2) and foraging and feeding in winter

Season	Behavior	Zoo	Edinburgh *N* = 47		Sapporo *N* = 69		Vienna *N* = 108	
			*Z*	*p*	*Z*	*p*	*Z*	*p*
Summer	Foraging	Debrecen	**−7.761**	**<.001**	−0.264	.792	**−10.856**	**<.001**
		Edinburgh	–	–	**−7.948**	**<.001**	**−6.618**	**<.001**
		Sapporo	–	–	–	–	**−11.176**	**<.001**
	Feeding	Debrecen	−1.409	.159	**−3.137**	**.002**	−0.956	.339
		Edinburgh	–	–	**−5.838**	**<.001**	**−3.524**	**<.001**
		Sapporo	–	–	–	–	**−5.745**	**<.001**
	Breeding	Debrecen	**−5.678**	**<.001**	**−6.346**	**<.001**	−2.18	.029
		Edinburgh	–	–	**−2.818**	**.005**	**−6.496**	**<.001**
		Sapporo	–	–	–	–	**−6.922**	**<.001**
Winter	Foraging	Debrecen	−1.568	.117	−0.944	.345	**−4.41**	**<.001**
		Edinburgh	–	–	−1.96	.050	**−5.747**	**<.001**
		Sapporo	–	–	–	–	**−4.93**	**<.001**
Winter	Feeding	Debrecen	−1.028	.304	**−4.541**	**<.001**	−1.617	.106
		Edinburgh	–	–	**−6.206**	**<.001**	**−5.19**	**<.001**
		Sapporo	–	–	–	–	**−5.293**	**<.001**

Note

Mann–Whitney tests, Bonferroni‐adjusted two‐tailed probabilities, significant (*p* < .008) differences are highlighted in bold.

Within‐zoo comparisons showed that feeding was the most frequently observed behavior of crows in Debrecen in the summer, and its frequency was higher than that of foraging (Figure 4A,C,E; *N* = 28, *Z* = −3.933, *p* < .001) or breeding (*N* = 30, *Z* = −3.951, *p* < .001). In the winter, however, foraging and feeding occurred in equal frequency (Figure 4B,D; *N* = 25, *Z* = −0.032, *p* = .983). In Edinburgh, foraging was the most frequently observed behavior and its frequency was higher than that of feeding both in the summer and winter (Figure 4; summer: *N* = 41, *Z* = −4.252, *p* < .001; winter: *N* = 39, *Z* = −2.477, *p* = .012), whereas the frequency of feeding and breeding behaviors did not differ (*N* = 42, *Z* = −2.04, *p* = .041, *α* = .016). In Sapporo, breeding behavior was more frequently observed than foraging (Figure 4A,C,E; *N* = 44, *Z* = −5.539, *p* < .001) or feeding (*N* = 52, *Z* = −5.097, *p* < .001) in the summer, whereas in the winter, foraging was more frequent than feeding (Figure 4B,D; *N* = 27, *Z* = −3.559, *p* < .001). In Vienna, the frequency of foraging was higher than that of feeding both in the summer (Figure 4; *N* = 108, *Z* = −9.022, *p* < .001) and the winter (*N* = 35, *Z* = −5.159, *p* < .001) and than that of breeding in the summer (*N* = 108, *Z* = −9.022, *p* < .001), when feeding was also more frequently observed than breeding (*N* = 87, *Z* = −6.793, *p* < .001).

## 4 DISCUSSION

Our study provides two main results. First, we found that the variability in crow numbers could be explained by season (in all zoos), time of day (in Debrecen, Sapporo, and Vienna), weather (in Debrecen and Vienna), and number of visitors (in Debrecen and Vienna). The number of crows increased two‐ to threefold from summer to winter in Debrecen, Edinburgh and Vienna likely by the influx of nonresident individuals. Further studies are required to discover where these nonresidents come from. In American Crows (*Corvus brachyrhynchos*), individuals appearing in urban sites in the winter are thought to come from rural populations, where nonterritorial floaters are opportunistic and do not show site fidelity, instead, they wander around in search of better foraging opportunities (Marzluff et al., 2001). Nevertheless, the influx clearly indicates the importance of zoos beyond providing breeding habitat to crows because the winter increase cannot be explained by breeding, which occurs only in the summer. Food availability is likely to be higher in the zoos than in natural environments, where food is scarce and/or under snow during the winter.

Time of day was important because we found more crows in the afternoon than in the morning in Debrecen, Sapporo, and Vienna. This finding may be explained by the temporal patterns of anthropogenic food sources. For example, birds may visit zoos to access leftover human food accumulating during the day in/near outdoor eateries, in trash bins, etc., as suggested by observations during the surveys in Vienna. In Debrecen, some zoo animals in wide open‐air enclosures are fed usually in the afternoon, and crows regularly appear in high numbers at this time, especially in the winter. Afternoons are probably important feeding times in the winter for crows because they need to collect energy to survive the cold nights at this time (Baltensperger et al., 2013). In Edinburgh, zoo birds and primates are fed twice a day (morning and afternoon), resulting in food potentially being available to crows all day, which may be related to the absence of a time‐of‐day effect here.

Weather was important for crow numbers in Debrecen and Vienna, with more crows observed in sunny weather than in cloudy weather with precipitation. It is likely that crows move around less when there is precipitation, for example, we observed that crows tended to stay longer in roosting sites in adverse weather and extreme cold in Sapporo, probably to save energy. In rainy weather, most crows stop feeding, become less active, and seek shelter from rain (Hume, 1986). Moreover, air uplift, which is necessary for crows for medium to long‐distance flight, does not occur on rainy days (Elkins, 1983). Although it is likely that crows taking refuge in trees were less detectable to observers than active crows in sunny weather, the differences in crow numbers were far greater than just a few individuals overlooked (Figure 4).

The number of visitors influenced crow numbers in Debrecen and Vienna similarly, with more crows when the number of visitors was few/intermediate, that is, 10 or less than when the number of visitors was many (over 10) in Debrecen, and with more crows with intermediate (6–10 visitors) than with few (5 or less) or many visitors in Vienna. This was expected because although crows can easily get used to the presence of humans in urban environments, they usually keep some distance from humans (Clucas & Marzluff, 2012; Matsyura, Jankowski, & Zimaroyeva, 2015) and thus are expected to avoid areas with too many visitors.

Our second main result was that we found significant variation in the distribution of foraging and feeding behaviors, which suggested that natural food sources were highly important for crows in Vienna (both seasons), less important in Edinburgh (both seasons) and in Sapporo (in winter only), and not important in Debrecen. In contrast, the importance of anthropogenic food sources was high in Debrecen (both seasons), lower in Edinburgh (both seasons), and lowest but still non‐negligible in Sapporo and Vienna. Observations during the surveys showed that foraging for natural food sources occurred almost exclusively in open areas (open ground, grasslands, lawns), and crows were hardly if ever seen foraging in wooded areas or forests. In contrast, feeding on anthropogenic food was typically observed near sources of such food, for example, outdoor eateries, kiosks, trash bins, and animal enclosures, and on trees where crows carried food items for further handling and consumption.

One likely reason for the relative importance of natural versus anthropogenic food sources may be related to the structure of habitats within the zoos (Figure 2, Table 1). In Edinburgh and Vienna, the zoos have extensive open, grassy areas, or parkland (Figure 2), which were favoured by crows for foraging. In Sapporo, much of the zoo area is built up or covered by trees and there are only small patches of open grassy area (Figure 2, Table 1). Finally, in Debrecen, the zoo area is almost completely covered by trees, and there are no open areas available for foraging for natural food inside the zoo (Figure 2). Extensive open areas lie just outside the Debrecen zoo, in a sport complex (Figure 2), where crows regularly forage for natural food both in the summer and winter. These differences in habitat structure provide a likely explanation for why foraging for natural food was frequently observed in Edinburgh and Vienna and feeding on anthropogenic food was frequent in Debrecen.

Sapporo zoo differed from the three zoos in Europe in that it had fewer crows and the number of crows decreased, rather than increased, from the summer to the winter. Possible reasons for this, beyond the relatively small open areas (see above), is that the zoo management imposed strict rules to constrain the use of the zoo area by free‐living birds. These actions were motivated by observations of crows stealing scraps of food directly from visitors. The majority of the animal enclosures are covered with net or wires to keep out crows (Table 1), and there are strict rules for the handling of zoo animal food. For example, large carnivores are given their food in the indoor parts of their pens. As a result, food availability is low and is mostly restricted to naturally occurring food. As a possible consequence of the scarcity of food, in the summer, the Sapporo zoo area is divided up into territories that are fiercely defended by territorial pairs of breeding crows. The crows observed in the zoo in the summer are, almost exclusively, resident breeding birds, which drive nonresidents out of the zoo area, which in turn explains the small and constant number of crows. During the surveys, for example, we observed territorial pairs defending their territories against flocks, sometimes of up to 250 and 300 crows that attempted to trespass or perch in their territories. In the winter, cold weather and snow cover reduce naturally occurring food so even the resident birds have to leave the zoo to find food elsewhere from time to time.

Our results on the importance of food sources do not refute the general observation that zoos provide ample opportunities for the breeding of the crows (Table 1). For example, in Vienna, as many as 45 active nests were found in 2012 within the zoo (C. Schwab, pers. obs.), corresponding to a high nesting density of 2.6 nests per hectare. This value is one magnitude higher than that reported anywhere else previously, for example, one such published maximum was 0.255 nests per hectare (Vuorisalo et al., 2003). High nesting densities may have important consequences on the social structure of crows through the emergence of colonial nesting, which is not expected in crows that traditionally nest solitarily in rural areas (Cramp & Perrins, 1994; McGowan, 2001). In Vienna, the social structure of crows shows an environmentally influenced fission–fusion dynamics centered around the zoo throughout the year (Uhl et al., 2018). In Debrecen, the center of establishment and colonization of crows in the city was the zoo (Kövér et al., 2015). Between 2006 and 2012, the city nesting population increased continuously, and the rate of increase was highest in the zoo area, which has many tall trees available for nesting. Here, nesting density increased from 2 to 8 nests/km^2^ in only seven years (Kövér et al., 2015). These previous results and our current results suggest that high availability of food and potential nesting sites are likely candidates to explain why crows are attracted to zoos.

We did not measure food availability directly, for example, by quantifying ground‐dwelling invertebrates or the amount of anthropogenic food accessible to crows. Rather, we used an indirect measure of food availability based on the behavior of the birds observed. In some cases, however, a higher frequency of foraging may not necessarily reflect higher availability of quality foraging areas with abundant natural food. For example, it is possible that crows are forced to forage for natural food rather than feed on anthropogenic food if the latter is not available, for example, by strict management of food for zoo animals, bird‐proof enclosures and garbage collection/storage, etc. Thus, foraging and feeding can be difficult to separate and more detailed information on natural and anthropogenic food sources, including their spatial and temporal variability, are necessary to disentangle the effects of natural versus anthropogenic food sources and the importance of the quantity versus quality of food to crows.

Our findings may have implications for the planning of urban areas and green infrastructure, as well as potential management implications for zoos regarding free‐living corvids, which may be especially relevant if crows become high in number. In many urban areas of the world, free‐living corvids come into conflict with humans (e.g., Soh, Sodhi, Seoh, & Brook, 2002), as large concentrations or high nesting densities of corvids may impact the soil and vegetation, decrease urban bird diversity, and present sanitary risks from the crows' use of waste dumps and leftovers for feeding (Matsyura, Zimaroyeva, & Jankowski, 2016; Zeller & Schuffenecker, 2004). The high importance of food, especially of anthropogenic food, in the attraction of crows to zoos suggests that appropriate management of such food sources could reduce the attractiveness of zoos to crows, if deemed necessary. Although crows are known for their intelligence in finding and utilizing various sources of food (Marzluff et al., 2008), our results and observations highlight several potential areas of intervention should they become necessary in zoos: (a) limiting human food consumption to restaurants in buildings inaccessible to crows, (b) if food for humans is served outdoors, regular cleaning of the premises and installing lockable, bird‐proof trash bins, (c) limiting or restricting zoo food that can be purchased and given to zoo animals by visitors, (d) imposing strict rules for the handling and provisioning of food to zoo animals by the zoo staff, and (e) covering open‐top enclosures with nets or wires. Our study provides an example for some of these interventions. In the case of Sapporo zoo, our results indicate that reducing the availability of anthropogenic food sources through these measures can be effective in decreasing the attractiveness of zoos to crows and in reducing crow numbers within the zoo. The number of crows was <5 per 10 ha in Sapporo zoo in both summer and winter (Figure 3A), even though the area of Sapporo zoo is c. 32% larger than that of the Debrecen and Vienna zoo (Table 1), which had more crows. Although we cannot exclude the possibility that biogeographic differences such as lower crow abundance in the general Sapporo region than in the regions of the other zoos can explain this difference, our experience with Sapporo zoo suggests that the emergence of territoriality and aggression toward nonresident conspecifics induced by low food availability (see above) can at least partly explain the lower crow numbers. The limitation of anthropogenic food was suggested by Preininger et al. (2019) as a solution to reduce the number of crows at waste disposal sites. Finally, in cases when crows need to be captured for research or for translocation purposes, zoos may provide suitable places for trapping crows, taking into account any relevant national legislation regarding crow trapping (Kövér, Tóth, Lengyel, & Juhász, 2018).

We conclude that crows are primarily attracted to zoos by food availability and secondarily by the availability of nesting sites. Both natural food and anthropogenic food contribute to increased food availability, and the relative importance of each appears to vary with habitat structure within and around the zoos. Our study thus draws attention to a previously overlooked role of zoos in the conservation of biodiversity (Conde, Flesness, Colchero, Jones, & Sceheurlein, 2011). It also provides useful information for the management of crow populations if they become high in number and for the planning of urban areas and green infrastructure.

## CONFLICT OF INTEREST

None declared.

## Data Availability

All data used in the analyses will available from Dryad upon acceptance. https://doi.org/10.5061/dryad.d2547d7zm
